# Comparison of Surgical Time, Short-term Adverse Events, and Implant Placement Accuracy Between Manual, Robotic-assisted, and Computer-navigated Total Hip Arthroplasty: A Network Meta-analysis of Randomized Controlled Trials

**DOI:** 10.5435/JAAOSGlobal-D-21-00200

**Published:** 2022-04-26

**Authors:** Kyle N. Kunze, Patawut Bovonratwet, Evan M. Polce, Katlynn Paul, Peter K. Sculco

**Affiliations:** From the Department of Orthopedic Surgery, Hospital for Special Surgery, New York, NY (Dr. Kunze, Dr. Bovonratwet, and Dr. Sculco), University of Wisconsin School of Medicine and Public Health, Madison, WI (Mr. Polce), and the Department of Orthopedic Surgery, Rush University Medical Center, Chicago, IL (Ms. Paul).

## Abstract

**Introduction:**

Recent years have observed the increasing utilization of robotic-assisted and computer navigation techniques in total hip arthroplasty (THA), given the proposed benefits of enhanced consistency and precision in implant placement. The purpose of this study was to conduct a systematic review of randomized controlled trials (RCTs) to determine whether differences in surgical times, adverse events, and implant positioning existed between manual, robotic-assisted, and computer navigation THA.

**Methods:**

PubMed, OVID/MEDLINE, and Cochrane databases were queried for RCTs comparing robotic-assisted versus manual THA and computer navigation versus manual THA at a minimum 1-year follow-up. Frequentist model network meta-analyses with P-scores were conducted to compare revisions, complications, and surgical times among the three treatment groups. A random-effects meta-analysis between computer navigation and manual THAs was conducted to analyze cup positioning because no robotic-assisted THA studies reported this outcome.

**Results:**

Five RCTs compared robotic-assisted and manual THAs, while seven compared computer navigation and manual THAs. manual THA was associated with significantly reduced surgical time in comparison with computer navigation (mean difference: 23.3 minutes) and robotic-assisted THAs (mean difference: 8.6 minutes; *P* < 0.001). No difference was observed in the incidence of all-cause complications (computer navigation: 1.7%, manual: 6.6%, and robotic-assisted: 16.2%) or revisions (computer navigation: 1.0%, manual: 1.7%, and robotic-assisted 4.8%) among the three treatment groups based on the network meta-analysis. In three studies that reported acetabular implant positioning, computer navigation had a significantly higher percentage of acetabular cups placed in the Lewinnek “safe zone” compared with manual THA (79% versus 52%; *P* = 0.02).

**Conclusions:**

manual THA results in markedly shorter surgical times and a similar incidence of complications and revisions compared with robotic-assisted and computer navigation THAs, given the sample sizes available for study. However, computer navigation THA led to increased precision in the placement of acetabular implants.

Total hip arthroplasty (THA) has been lauded as one of the most successful procedures within orthopaedic surgery because it confers reproducible and clinically significant improvements in pain, function, and quality of life.^1,2,3,4,5^ As the demand for THA within the United States continues to rise annually, it will be imperative to avoid sacrificing the quality of patient outcomes for increases in surgical volume.^6^ Although there are numerous factors that contribute to clinical outcomes and patient satisfaction after THA, avoidance of complications is paramount to a successful outcome.

To enhance the reproducibility of THA and avoid complications such as implant malpositioning, dislocation, and bearing wear, orthopaedic surgeons have embraced the use of robotic and computer navigation technologies. The proposed benefits of robotic-assisted and computer navigation THAs include increased precision and accuracy of the acetabular cup version and inclination along with more consistent restoration of leg length and offset.^7,8,9,10^ However, some studies claim that there is little benefit to robotic-assisted or computer navigation THAs when compared with manual THA^11,12^ because this method may result in equivalent outcomes at the expense of increased surgical time and substantial cost.^13^ Therefore, a better understanding of the cost-benefit relationship between the utilization of robotics and computer navigation and patient outcomes after THA is important to evaluate.

Although previous literature has sought to investigate the influence of manual versus robotic-assisted and computer navigation THAs on patient outcomes, these studies have been limited by the level of evidence of included articles. The purpose of this study was to conduct a systematic review of only level 1 evidence to determine whether there are any differences in surgical time, adverse events, and implant positioning between manual, robotic-assisted, and computer-navigated THAs.

## Methods

### Search Process

This systematic review and meta-analysis was conducted in accordance with the 2009 Preferred Reporting Items for Systematic Review and Meta-Analysis guidelines.^14^ The query for studies was done in August 2020 using the Cochrane Database of Systematic Reviews, the Cochrane Central Register of Controlled Trials, PubMed (2008 to 2019), and OVID/MEDLINE (2008 to 2019) databases using the Boolean search strategy “(((((Hip) AND (arthroplasty)) OR (THA)) AND (robotic))) OR (computer-nav*).”

### Selection Process

Inclusion criteria for the search mentioned earlier consisted of all studies published in the English language reporting information regarding the use of robotic-assisted or computer-navigated THAs. Exclusion criteria consisted of (1) nonrandomized controlled trials, (2) cadaveric studies, (3) animal studies, (4) preclinical articles, (5) editorial articles, (6) surveys or case reports, and (7) less than 1-year minimum follow-up. Two investigators independently reviewed the abstracts from all identified articles. Full-text articles were obtained for review to allow additional assessment of inclusion and exclusion criteria when necessary. Bibliographies of relevant systematic reviews and included articles were manually searched for additional references.

### Quality Assessment Measures

The Jadad Scale^15^ was used to assess the methodological quality of all included randomized controlled trials (RCTs). The Jadad Scale consists of a five-point questionnaire used to critically evaluate the methodological quality of RCTs. The following questions are used to assess each study: (1) Was the study described as random? (2) Was the randomization scheme described and appropriate? (3) Was the study described as double-blind? (4) Was the method of double blinding appropriate? (5) Was there a description of dropouts and withdrawals? The scale is graded from 0 to 5 (a score of greater than or equal to 3 indicates a high-quality study, whereas a score less than 3 is considered to be low-quality). The interobserver reliability for the two independent graders (blinded for review) was excellent at 0.95 (95% confidence interval, 0.91 to 0.99).

### Statistical Analysis

Categories for data collection for each full article included (1) publication characteristics, (2) patient demographics, (3) patient-reported outcome measures, (4) adverse events, (5) inclusion and exclusion criteria; (6) and implant positioning data, including acetabular inclination and version, and proportion of acetabular cups placed within the safe zone. Means and standard deviations were extracted to calculate pooled values for patient demographics. Ranges were reported for all outcomes not amenable to meta-analysis to avoid improper pooling and misinterpretation bias. Owing to variability in the reporting of patient-reported outcome measures, these were not considered for the meta-analysis. The Fisher exact test was used to compare specific revision and complications among the three cohorts.

A frequentist network meta-analysis model was used to compare the effect of robotic-assisted, computer navigation, and manual THAs on the outcomes of surgical time, revision surgeries, and postoperative complications.^16^ The assumption of consistency from direct and indirect evidence under the random-effects model was assessed using the Q statistic.^17^ Competing treatments were ranked in the network meta-analysis by using point estimates and standard errors to calculate P-scores.^18^ P-scores represent a measure of certainty that a specific treatment is superior to other treatments in the network model for a given outcome. The higher the P-score for a given treatment, the more certainty the treatment is superior in comparison with the other treatments. We used the term “treatment ranking” to order the treatment arms by P-score from best to worst treatment for each outcome.

Given that a network meta-analysis compares multiple treatments, these models consider both direct (comparisons from studies that include both treatment arms) and indirect evidence (comparisons that did not include both treatment arms within a study). For example, if a study compared complications between manual and robotic THAs (direct evidence), the network model also considers manual THA complications in studies that compare manual and computer navigation THAs, although these contribute less weight to the overall effect because indirect evidence constitutes a smaller proportion of the total evidence. Therefore, although the cumulative number of an event (complications) may seem markedly greater in one treatment arm compared with another, the events in the subset of studies providing direct evidence in comparing two treatment arms may be similar and contribute more weight.

Precision of implant positioning was analyzed through a random effects meta-analysis of the pooled rate of acetabular cup placement in the Lewinnek safe zone.^19^ Network meta-analysis could not be conducted for this outcome, given that none of the included studies concerning robotic-assisted THA assessed implant positioning. Therefore, only computer navigation–assisted and manual THAs were compared for this outcome. Forest plots were used to graphically depict the comparison of surgical time, implant positioning, and complication and revision surgery rates between competing treatments. Study heterogeneity was assessed using I-squared (I^2^) tests. All statistical analyses were conducted using R Project for Statistical Computing software (RStudio software version 1.2.1335; R Foundation for Statistical Computing).

## Results

### Study Characteristics and Descriptions

**Figure 1:**
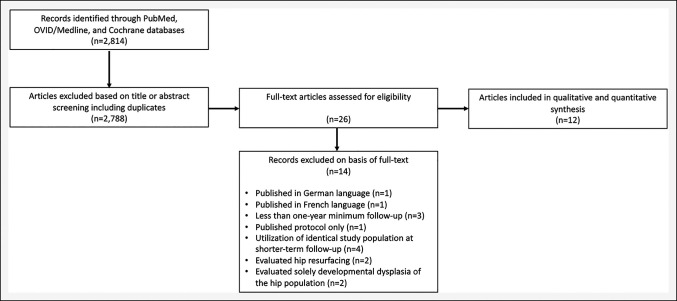
Flowchart showing Preferred Reporting Items for Systematic Review and Meta-Analysis guidelines for included studies.

A total of 12 RCTs that included 1,139 patients were included in the final analysis (Figure 1). Seven of these studies^20,21,22,23,24,25,26^ assessed the use of computer-navigated THA versus manual THA while the remaining five^27,28,29,30,31^ assessed the use of robotic-assisted THA versus manual THA. The mean age (robotic: 59.5 versus computer navigation: 61.2 versus manual: 61.0, *P* = 0.90) and BMI (robotic: 25.7 versus computer navigation: 27.0 versus manual: 26.8 kg/m^2^, *P* = 0.83) did not differ between the three cohorts. The pooled mean follow-up was 4.3 years (range, 1 to 14.2 years) among all 12 studies, and there was no difference in the mean follow-up between the groups (robotics versus manual THA 6.3 years and computer navigation versus manual THA 2.8 years; *P* = 0.21). All robotic-assisted THA were done with the ROBODOC/ORTHODOC (Integrated Surgical Systems) system for femoral preparation and milling (Table 1), while a wide variety of computer-navigated (Table 2) systems were used. Therefore, no RCTs investigating robotic-assisted THA used systems that apply robotic guidance for acetabular implant placement.

**Table 1:** Studies Using Robotic-assisted Total Hip Arthroplasty

Author, Year	Patients (manual)	Patients (Robotic)	Robotic System	Inclusion Criteria	Exclusion Criteria	Approach
Honl et al, 2003^31^	80	61	ROBODOC/ORTHODOC	Hip OA	Not reported	Anterolateral
Nishihara et al, 2006^30^	78	78	ROBODOC/ORTHODOC	Good bone quality (Dorr type A or B) and Crowe class I-III.	Poor bone quality (Dorr type C) and Crowe class IV	Posterolateral
Lim et al, 2015^28^	25	24	ROBODOC/ORTHODOC	Ages 20-70 yrs and willingness to participate in follow-ups	Age older than 70 yrs, inadequate femoral bone stock (Dorr type C), septic hip sequelae, Crowe type III-IV, and fused hip	Not reported
Nakamura et al, 2010^9^	64	64	ROBODOC/ORTHODOC	Hip OA, Dorr A/B, and Crowe I-III	Not reported	Posterolateral
Bargar et al, 2018^27^	22	45	ROBODOC/ORTHODOC	Not reported	Not reported	Posterolateral

OA = osteoarthritis

**Table 2:** Studies Using Computer Navigation-assisted Total Hip Arthroplasty

Author, Year	Patients (manual)	Patients (CN)	Imaging/Imageless	Navigation/Imaging System (Focus)^a^	Inclusion Criteria	Exclusion Criteria	Approach
Leenders et al, 2002^21^	50	50	Imaging	SurgiGate (cup position)	Primary THA	Not reported	Anterolateral
Gurgel et al, 2014^23^	20	20	Imageless	OrthoPilot (cup position)	Primary hip OA or osteonecrosis, age 20-80 yrs, and BMI <35 kg/m^2^	Refusal to participate, serious illness that would make surgery impossible, hip dysplasia, any previous orthopaedic surgery excluding contralateral THA, and patient death.	Direct lateral
Lass et al, 2014^22^	63	62	Imageless	NaviTrack (cup position, +FL, and +FO)	Primary/posttraumatic OA or osteonecrosis, and age >18 yrs	Revision THA, prior pelvic/trochanteric osteotomy, and suspicion for infection	Modified transgluteal
Renkawitz et al, 2015^24^	69	66	Imageless	BrainLAB Prototype Hip 6.0 with Femur First (cup position and calculation of an impingement-free zone)	Primary/secondary OA, age 50-75 yrs, and ASA ≤3	Age <50 yrs or >75 yrs, ASA >3, hip dysplasia, posttraumatic OA, and previous hip surgery	Anterolateral
Parratte et al, 2016^25^	30	30	Imageless	Adaptation of Hiplogics Praxim Medivision (cup position)	Primary THA, age 20-80 yrs, weight <100 kg, a anterolateral approach, and procedure performed by the senior author	Revision THA, concomitant trochanteric osteotomy, and posttraumatic OA	Modified Watson-Jones anterolateral approach
Weber et al, 2016^26^	32	28	Imageless	BrainLAB Prototype Hip 6.0 with Femur First (cup position, calculation of an impingement-free zone)	Primary THA, age 50-75 yrs	Hip dysplasia	Not reported
Verdier et al, 2016^20^	39	39	Imaging	NAVEOS EOS (cup position)	Primary/secondary hip OA without subluxation	Revision THA, hip dysplasia, and inability to give informed consent	Anterolateral

ASA = American Society of Anesthesiologists score, BMI = body mass index, computed tomographyCN = computer navigation, +FL = system provides feedback on leg length, +FO = system provides feedback on offset, OA = osteoarthritis, THA = total hip arthroplasty

a Focus: The primary function of the navigation system for planning and optimization.

### Surgical Time

**Figure 2:**
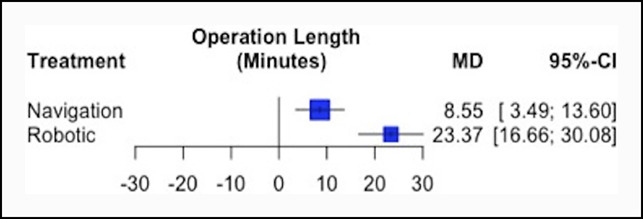
Forest plot showing the standardized MD and 95% CI in operation length in minutes for robotic and navigation-assisted THAs relative to manual THA. CI = confidence interval, MD = mean difference, THA = total hip arthroplasty.

Of the 12 RCTs, six reported surgical time and were included in the network meta-analysis. Three studies (N = 346 patients) directly compared robotic and manual THAs, and three studies (N = 373 patients) compared navigation-assisted and manual THAs. Conducting manual THA (pooled mean: 86.6 minutes) was associated with a significantly reduced mean surgical time in comparison with both robotic-assisted (pooled mean: 110.7 minutes) and navigation-assisted THAs (pooled mean: 95.7 minutes; *P* < 0.001) (Table 3 and Figure 2). Navigation-assisted THA was also found to have a significantly reduced surgical time in comparison with robotic-assisted THA (*P* < 0.001). Treatment rankings (ordered from the best to worse THA method for surgical time) based on the network meta-analysis were as follows: (1) manual (P-score = 1.00), (2) navigation-assisted (P-score = 0.50), and (3) robotic (P-score = 0.00).

**Table 3:** Pairwise Comparison of THA Surgical Treatments in the Network Meta-Analysis

Outcome	Treatment Comparison	OR Estimate (95% CI)	*P* Value
Complications			
	Robotic versus manual	1.61 (0.68-3.83)	0.281
	Navigation versus manual	0.83 (0.23-2.99)	0.781
	Robotic versus navigation	1.93 (0.41-9.02)	0.403
Revisions			
	Robotic versus manual	1.11 (0.38-3.23)	0.845
	Navigation versus manual	1.15 (0.30-4.42)	0.840
	Robotic versus navigation	0.97 (0.17-5.39)	0.970
		SMD estimate (95% CI)	
Operation length			
	Robotic versus manual	23.37 (16.66-30.08)	**<0.001**
	Navigation versus manual	8.55 (3.49-13.60)	**<0.001**
	Robotic versus navigation	14.82 (6.43-23.22)	**<0.001**

CI = confidence interval, OR = odds ratio, SMD = standardized mean difference, THA = total hip arthroplasty

Reference group is listed second in each respective row of the treatment comparison column.

Bold values indicate statistical significance (*P* < 0.05).

### Revision Rates

**Figure 3:**
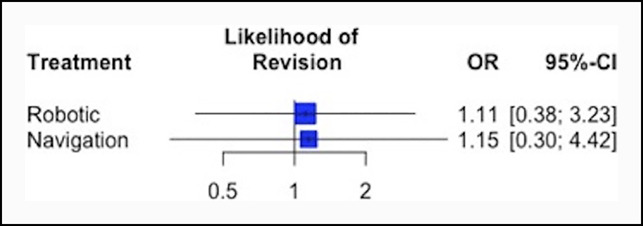
Forest plot showing the OR and 95% CI of revisions for robotic and navigation-assisted THA relative to manual THA. CI = confidence interval, OR = odds ratio, THA = total hip arthroplasty.

Revision rates were reported in all 12 studies, with five studies (N = 541 patients) directly comparing robotic-assisted THA with manual THA and seven studies (N = 598 patients) comparing navigation-assisted THA with manual THA. No significant differences were observed between treatments for all-cause postoperative revisions with the sample sizes available for study (Table 4 and Figure 3). Treatment rankings (ordered from the best to worse THA method for incidence of all-cause revisions) based on the network meta-analysis were as follows: (1) manual (P-score = 0.58), (2) robotic (P-score = 0.47), and (3) navigation-assisted (P-score = 0.45).

**Table 4:** Revision and Complication Rates and Events Among Studies Stratified by the THA Approach

Outcome	Robotic	Computer Navigation	Manual^a^	Manual^b^	Manual^a,b^
Revisions	Total = 13 Head and liner change secondary to polyethylene wear (N = 3) Periprosthetic fracture (N = 1) Heterotopic ossification (N = 1) Dislocation (N = 5) Severe Trendelenburg gait (N = 3)	Total = 3 Infection (N = 1) Periprosthetic fracture (N = 1) Dislocation (N = 1)	Total = 8 H&L change secondary to polyethylene wear (N = 5) Infection (N = 2) Periprosthetic fracture (N = 1)	Total = 2 Periprosthetic fracture (N = 2)	Total = 10 H&L change secondary to polyethylene wear (N = 5) Infection (N = 2) Periprosthetic fracture (N = 3)
Complication	Total = 44 DVT (N = 4) Nerve palsy (N = 4) Dislocation (N = 12) Heterotopic ossification (N = 20) Wound dehiscence/issue (N = 4)	Total = 5 Femur fractures/cracks (N = 3) Dislocation (N = 1) Infection (N = 1)	Total = 31 DVT (N = 3) Femur fractures/cracks (N = 7) Nerve palsy (N = 1) Dislocation (N = 3) Heterotopic ossification (N = 12) Infections (N = 2) Wound dehiscence/issue (N = 3)	Total = 7 Femur fractures/cracks (N = 3) Death (N = 1) Nerve palsy (N = 1) Dislocation (N = 1) Infection (N = 1)	Total = 38 DVT (N = 3) Femur fractures/cracks (N = 10) Death (N = 1) Nerve palsy (N = 2) Dislocation (N = 4) Heterotopic ossification (N = 12) Infection (N = 3) Wound dehiscence/issue (N = 3)

DVT = deep vein thrombosis, H&L = head and liner, THA = total hip arthroplasty

a manual treatment arms in robotic studies.

b manual treatment arms in navigation studies.

The most common reason for revision in the manual cohort was head and liner exchange for polyethylene wear (n = 5), while it was dislocation (n = 5) for the robotic-assisted cohort. The three revisions in the computer navigation cohort were for periprosthetic joint infection (n = 1), dislocation (n = 1), and periprosthetic fracture (n = 1). When comparing revisions for instability between the groups, five patients (1.8%) were revised in the robotic-assisted cohort, one (0.34%) was revised in the computer navigation cohort, and none were revised in the manual cohort (*P* = 0.002). Post hoc analysis revealed that robotic THA was associated with more revisions for instability compared with manual THA (*P* = 0.003) but not computer-navigated THA (*P* = 0.11). Furthermore, there was no significant difference between the rate of revision for instability between the manual and computer navigation cohorts (*P* = 0.34). No association was observed between the THA method and the head and liner exchange for polyethylene wear (*P* = 0.19), with three patients (1.1%) revised with a head and liner exchange in the robotic cohort, zero patients (0.0%) in the computer navigation cohort, and five patients (0.87%) in the manual cohort (Table 4).

### Complication Rates

**Figure 4:**
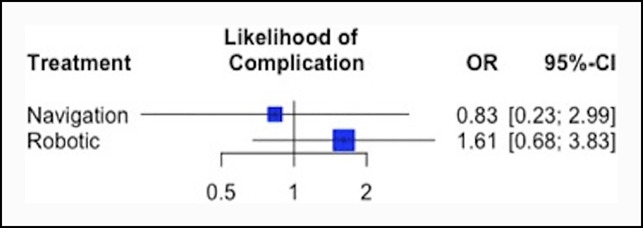
Forest plot showing the OR and 95% CI of postoperative complication for robotic and navigation-assisted THA relative to manual THA. CI = confidence interval, OR = odds ratio, THA = total hip arthroplasty.

Postoperative complication rates were reported in a total of 11 studies. No significant differences were observed between the three groups for likelihood of all-cause postoperative complications (Table 4 and Figure 4) based on the network meta-analysis, with 5 complications (1.7%) in the computer navigation cohorts, 44 complications (16.2%) in the robotic-assisted THA cohort, and 38 complications (6.6%) in the manual THA cohort. Treatment rankings (ordered from the best to worse THA method for incidence of all-cause complications) based on the network meta-analysis were as follows: (1) navigation-assisted (P-score = 0.70), (2) manual (P-score = 0.63), and (3) robotic (P-score = 0.17). Robotic-assisted THA was associated with more postoperative dislocations (n = 12, 4.4%) compared with computer navigation (n = 1, 0.34%) and manual THAs (n = 4, 0.7%) (*P* < 0.001). However, there was no significant difference is dislocation rates between the computer navigation and manual cohorts (*P* = 0.67).

### Implant Positioning

**Figure 5:**
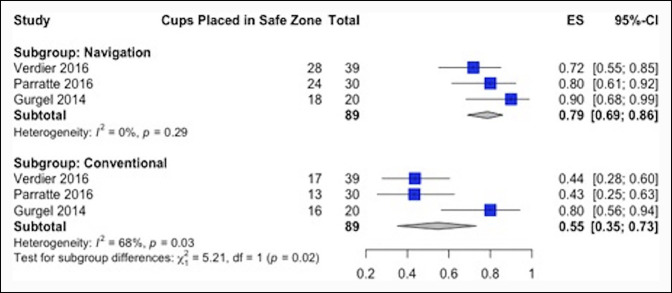
Forest plot showing the proportion of acetabular cups placed in the Lewinnek safe zone stratified by the treatment group. It shows ES (the safe zone placement rate represented by squares, proportional in size to the sample size of each study) and confidence intervals (horizontal lines). ES = effect size, CI = confidence interval, I^2^ = I-squared.

The number of acetabular cups placed in the Lewinnek safe zone relative to the total number of cups placed (Figure 5) was reported in three studies for both computer navigation (N = 89 patients) and manual THAs (N = 89 patients). One study reported only target inclination about the Lewinnek value (inclination 40° ± 10°) and therefore was not included in the meta-analysis. The use of computer navigation THA was associated with a significantly higher percentage of acetabular cups placed in the safe zone compared with manual THA (70/89, 79% versus 46/89, 52%, *P* = 0.02). Moderate heterogeneity was observed between pooled studies in the manual THA cohort (I^2^ = 68%), whereas the computer navigation THA cohort had no statistical heterogeneity (I^2^ = 0%). No studies comparing robotic-assisted and manual THAs reported on acetabular cup positioning.

### Methodological Quality Assessment

The mean Jadad score among all 12 studies was 3.6 ± 1.1 (Table 5), indicating high-quality study methodology on average. The Jadad score ranged from 2 to 5, with a total of four studies (33.3%) scoring a 5/5 and indicating no methodological flaws in their RCT study designs and reporting. The mean Jadad score for the robotic studies was 2.8 ± 0.4, while the mean Jadad score for the computer navigation studies was 3.7 ± 1.3 (*P* = 0.15).

**Table 5:** Jadad Scores for Included Studies

Author, Year	Jadad Score
Robotic	
Honl et al, 2003^31^	2
Nishihara et al, 2006^30^	3
Lim et al, 2015^28^	3
Nakamura et al, 2010^9^	3
Bargar et al, 2018^27^	3
Computer navigation	
Leenders et al, 2002^21^	3
Gurgel et al, 2014^23^	3
Lass et al, 2014^22^	3
Renkawitz et al, 2015^24^	5
Parratte et al, 2016^25^	5
Weber et al, 2016^26^	5
Verdier et al, 2016^20^	2

## Discussion

Robotic-assisted and computer-navigated THAs have become increasingly popular with a purported benefit of more precise implant positioning that could lead to a reduced risk of complications. However, with the emphasis on value-based care, questions remain whether the costs of these procedures outweigh these proposed benefits. The results from this study demonstrate that manual THA leads to reduced operating room time when compared with computer navigation–assisted and robotic THAs, with no significant differences in rates of all-cause postoperative complications or revisions. However, robotic-assisted THA was associated with a higher incidence of postoperative dislocations and revisions for instability, although the only robotic systems implicated in RCTs to date and subsequently identified in this systematic review used robotic assistance only for preparation of the femoral implant. The use of computer navigation led to more precise positioning of acetabular implants and trends toward fewer complications.

The increased surgical time with robotic-assisted and computer-navigated THAs found in our study corroborates findings found previously. Specifically, we found that manual THA resulted in a mean 8.6-minute shorter procedure compared with computer navigation THA and a 23.4-minute shorter procedure compared with robotic THA. Liu et al^32^ conducted a systematic review of seven computer navigation versus manual THA studies and reported that surgical times were significantly increased for the computer navigation method (mean difference, 23.0 minutes, *P* < 0.001). Other systematic reviews solely comparing computer navigation and manual THAs^33,34^ or robotic-assisted and manual THAs^35^ have also reported increased surgical times with these approaches compared with manual THA. The increased surgical time is likely because of the learning curve encountered when using these new technologies and the setup required, which is greater than that of manual THA. However, previous studies have indicated that over time with the continued use of robotics or computer-navigated THA, surgical times gradually decrease and may become comparable with manual THA.^36,37^ Future studies are warranted to determine which factors associated with these procedures contribute the most to the increased surgical times found in multiple studies.

This study demonstrated that there are no differences in the incidence of all-cause complications or all-cause revision THA when comparing the three techniques. However, there was a trend toward an increased incidence of all-cause complications with manual THA when compared with computer-navigated THA. Montgomery et al^12^ conducted a Medicare database analysis of 64,944 THA procedures (5,412 computer navigation THA versus 59,532 manual THA) and reported that computer navigation THA and manual THA had comparable dislocation rates, but computer navigation resulted in markedly higher rates of revision THA at 30 days (1.0% versus 1.4%) and 90 days (1.2% versus 1.7%); however, these were 0.4% and 0.5% differences, respectively. However, our data are more consistent with those reported by Bohl et al.^38^ In a large claims database of claims database of 803,732 primary THA procedures, Bohl et al found that navigation-based THA was associated with reductions in rates of dislocation and aseptic acetabular revisions compared with manual THA. Furthermore, a recent nationwide database study of 309,252 THAs done by Gausden et al^39^ found that the use of computer-navigated THA resulted in a 12% reduced odds of 90-day complications and lower readmission rates for dislocation in comparison with manual THA. In this study, the complication rate for computer navigation THA was 0.7%, whereas manual THA had a complication rate of 1.4%. There were no acetabular implant revisions for loosening in either cohort, which may be a function of the follow-up period; however, only one dislocation was experienced in the computer navigation cohort, whereas four were experienced in the manual THA cohort. Furthermore, a total of five patients who underwent manual THA subsequently went on to have head and liner exchanges for excessive wear, while no patients in the computer navigation cohort had revisions for this reason. Although it is logical that navigation-based THA would result in fewer acetabular implant revisions because it is theorized to improve cup placement, we are limited in our ability to draw conclusions from these findings with these data, given that the head and liner exchanges came from one study in which patients underwent THA between 1994 and 1998 and therefore highly cross-linked polyethylene was not used. However, our findings remain supported by the previous literature discussed earlier.

The results from this study demonstrate that robotic-assisted THA was associated with a markedly higher rate of postoperative dislocations and revision for instability when compared directly. However, it should be noted that these robotic systems only used this technology for preparation and milling of the femoral canal. To our knowledge, there are currently no RCTs using more contemporary robotic systems. Therefore, this may have influenced the increased dislocation and revision rates observed among these studies. Despite the fact that the acetabular implant was placed manually, one may still expect to observe no significant differences in dislocations and revisions for instability when compared with the manual THA cohort. Interestingly, a recent meta-analysis of seven robotic-assisted versus manual THA studies reported that the odds ratio for postoperative complications for robotic-assisted THA compared with manual THA was 3.35 (95% confidence internal, 0.94 to 11.91), although this did not reach statistical significance (*P* = 0.06).^35^ However, these authors also reported that (1) the rate of dislocations was higher in the robotic cohort than the manual cohort, (2) the rate of revision was slightly higher in robotic patients (two versus one) and was attributed to abductor insufficiency, and (3) one included study noted a significantly higher rate of Trendelenburg signs postoperatively in robotic patients (61% versus 26%, *P* = 0.0014). Although conflicting evidence exists among lower quality systematic reviews and trials, this study of RCTs indicates no difference in all-cause complication and revision rates among these treatments using a network approach; however, it seems that there is an association between the robotic-assisted THA approach and the resultant instability and dislocations when conducting direct comparisons that do not account for study variability because statistically significant differences were observed. In addition, the overall complication rate of robotic THA was high at 16.2%, the significance of which may not be clear using a network approach. Future RCTs are required to examine the short-term complications of systems that implicate robotic assistance for both acetabular and femoral implant placements and that focus on direct comparisons of these adverse events.

Computer navigation THA resulted in markedly better acetabular cup precision and accuracy when compared with manual THA. Specifically, a total of 79% of the cups were placed in the Lewinnek safe zone with computer-assisted THA compared with 52% of the cups with manual THA. A recent systematic review also reported statistically significant improvements in precision and accuracy for computer navigation cup placement when compared with manual THA.^40^ Although no RCTs assessed acetabular cup placement as an outcome, Domb et al^7^ found that 100% of the robotic-assisted THA acetabular cups were placed within the Lewinnek safe zone compared with only 80% of the manual THA acetabular cups (*P* = 0.001). Therefore, it is possible that the same benefits observed for computer navigation THA in this study are also applicable for robotic-assisted THA. Indeed, previous literature has suggested this equivalency, although it remains limited. Ando et al^41^ compared robotic-assisted THA with navigation-based THA for patients with osteoarthritis secondary to hip dysplasia and reported an advantage for robotic THA in reducing acetabular cup placement variation, although the clinical relevance of this finding was not clear. Shibanumaet al^42^ reported no statistically significant differences in postoperative radiographic inclination or anteversion between robotic-assisted versus navigation-assisted THA in a prospective study involving 60 patients. Overall, although meaningful comparisons between these two methods were not available given in the study data, comparison between computer navigation THA and manual THA demonstrated that computer navigation THA confers superior accuracy and precision of acetabular cup placement without jeopardizing safety as measured by complications and revisions as compared with manual THA. This finding may also have accounted for the absence of acetabular cup revisions in this cohort (Table 4). However, future randomized studies are warranted to determine whether this statement holds true for robotic-assisted THA when compared with manual THA and whether these differences in surgical times and cup placement are clinically important.

Recent literature, which has used Markov modeling for both robotic-assisted and computer-navigated THAs, has reported that potential reductions in revision rates, complications, and readmissions make these systems cost-effective compared with manual THA.^43^ Because these models depend on retrospectively collected data for recently available systems, studies included in this review may not reflect the cost-effectiveness associated with these findings. This review highlights the need for future RCTs to study a wider range of robotic systems that are currently available that assist with both acetabular and femoral preparation because the current data suggest that robotic preparation of solely the femoral implant may result in costly complications and revisions related to the acetabular preparation. Future studies are needed to compare the immediate and long-term cost differences between these three studies, if they exist, should the data become available.

The results of this study should be considered in the context of several limitations. First, as with all systematic reviews, publication bias may be present, which may influence our results because studies with equivocal findings may be less likely to be published and therefore would not be included in this review. Second, owing to variable reporting and outcome heterogeneity, we were unable to conduct a network meta-analysis of acetabular implant positioning and patient-reported outcome measures. Third, we used the Lewinnek safe zone as the benchmark for proper implant positioning. We recognize that this may not be the best position for an acetabular implant for all patients, particularly those with spinopelvic pathology such as a stiff lumbar spine. However, we do not think that this greatly limits our comparison of precision between the three techniques because most of the included studies were published before the emphasis on the spinopelvic relationship and acetabular implant positioning. Fourth, we recognize that the only published RCTs to date on robotic-assisted THA evaluate one system. This system only used robotic assistance for preparation and milling of the femoral canal, and the acetabular implant was placed manually, which limits our ability to draw conclusions on the influence of robotics on the placement of the acetabular implant. In addition, given that only one robotic system was used, we are thus unable to draw any conclusions regarding other robotic systems that may be in the market. Fifth, 10 different computer navigation systems were used in the included studies. We are unable to draw any conclusions regarding any one particular system based on the results from this study. To draw any conclusions regarding any particular computer navigation or robotic system requires multiple independently funded, bias-free, well-designed, prospective RCTs with long-term follow-up of both clinical and radiographic outcomes. Finally, we did not exclude studies based on the date of publication or when THA was done. This is relevant because several studies included THAs done before the adoption of highly cross-linked polyethylene. This in addition to the long follow-up likely explains why all the head and liner exchanges included in the manual cohort came from one study with a mean 14.2-year follow-up in which the patients underwent THA between 1994 and 1998.

## Conclusion

Based on currently available level 1 RCTs, manual THA results in significantly shorter surgical times and a similar incidence of complications and revisions compared with RA and CN THAs, given the sample sizes available for study. However, CN THA led to increased precision in the placement of acetabular implants in the “safe zone” compared with manual THA.
